# Nucleus basalis of Meynert revisited: anatomy, history and differential involvement in Alzheimer’s and Parkinson’s disease

**DOI:** 10.1007/s00401-015-1392-5

**Published:** 2015-01-30

**Authors:** Alan King Lun Liu, Raymond Chuen-Chung Chang, Ronald K. B. Pearce, Steve M. Gentleman

**Affiliations:** 1 Neuropathology Unit, Division of Brain Sciences, Department of Medicine, Imperial College London, London, UK; 2 Laboratory of Neurodegenerative Diseases, Department of Anatomy, LKS Faculty of Medicine, The University of Hong Kong, Hong Kong S.A.R., China; 3 State Key Laboratory of Brain and Cognitive Sciences, The University of Hong Kong, Hong Kong S.A.R., China; 4 Research Centre of Heart, Brain, Hormone and Healthy Aging, LKS Faculty of Medicine, The University of Hong Kong, Hong Kong S.A.R., China

**Keywords:** Nucleus basalis of Meynert, Topography, Parkinson’s disease, Alzheimer’s disease, Neuropathology

## Abstract

It has been well established that neuronal loss within the cholinergic nucleus basalis of Meynert (nbM) correlates with cognitive decline in dementing disorders such as Alzheimer’s disease (AD). Friedrich Lewy first observed his eponymous inclusion bodies in the nbM of postmortem brain tissue from patients with Parkinson’s disease (PD) and cell loss in this area can be at least as extensive as that seen in AD. There has been confusion with regard to the terminology and exact localisation of the nbM within the human basal forebrain for decades due to the diffuse and broad structure of this “nucleus”. Also, while topographical projections from the nbM have been mapped out in subhuman primates, no direct clinicopathological correlations between subregional nbM and cortical pathology and specific cognitive profile decline have been performed in human tissue. Here, we review the evolution of the term nbM and the importance of standardised nbM sampling for neuropathological studies. Extensive review of the literature suggests that there is a caudorostral pattern of neuronal loss within the nbM in AD brains. However, the findings in PD are less clear due to the limited number of studies performed. Given the differing neuropsychiatric and cognitive deficits in Lewy body-associated dementias (PD dementia and dementia with Lewy bodies) as compared to AD, we hypothesise that a different pattern of neuronal loss will be found in the nbM of Lewy body disease brains. Understanding the functional significance of the subregions of the nbM could prove important in elucidating the pathogenesis of dementia in PD.

## Introduction

Although the identification of Lewy bodies (LB) and neuronal loss in the substantia nigra is considered the gold standard for the neuropathological diagnosis of Parkinson’s disease (PD), these two pathological features were actually first recognised by Friedrich Lewy in the nucleus basalis of Meynert (nbM) in 1913. Within the basal forebrain sublenticular region, there is a broad band of cell clusters commonly known as the nbM. Neuronal loss in the nbM is well established in dementing disorders; however, its pathological significance was first recognised in a series of patients with *paralysis agitans* (now known as PD) by Lewy where severe neuronal degeneration and intraneuronal globose tangles were noted [63]. He also observed that concentric hyaline-rich “*Kugeln*” (balls, as originally identified in the globus pallidus) were found in surviving neurons in the nbM and dorsal motor nucleus of the vagus [89]. These intraneuronal inclusions were later given the name LB and the presence of LB became one of the cardinal neuropathological features of PD. Lewy speculated that the nbM neuronal loss was responsible for some of the motor deficits seen in PD and it was not until the 1930s that Hassler suggested the pathological changes in the nbM were probably related to cognitive function deficits in PD (in [94]). Subsequently, the nbM has been investigated extensively in many neuropsychiatric disorders including schizophrenia [4, 103], Pick’s disease [86], Alzheimer’s disease (AD) [2, 4–6, 17, 19, 25, 29, 46, 47, 64, 65, 73, 74, 78–81, 85, 86, 94, 100–102, 105], Creutzfeldt–Jakob disease [86], dementia pugilistica [96] and Down’s syndrome [18, 86]. The functional significance and connections of the nbM were unknown until the 1970s when the cholinergic hypothesis in AD was proposed [9]. The nbM was then found to be a cholinergic centre, with neurons providing cholinergic afferents to the entire neocortex [24, 55, 67, 69]. Hence, the decrease in cortical acetylcholine levels seen in dementing disorders was thought to relate to cell death within the nbM.

The nbM is a broad and irregular “nucleus” in the human forebrain and functional subdivision of the nbM has been suggested, based on the topographical projection of cholinergic fibres from the nbM in non-human primates [69]. However, this topography is not directly translatable to the human brain. It is, therefore, important to revisit this question of anatomical subdivision of the nbM for the investigation of possible clinicopathological correlations in different dementias.

With the advances in imaging of the basal forebrain, and the nbM potentially being the next target for neuromodulation with deep brain stimulation (DBS) [37], we will review the history of the localisation of this basal forebrain nucleus and look at possible trends in clinicopathological correlation of different nbM subsectors.

## Where exactly is the nbM?

**Fig. 1:**
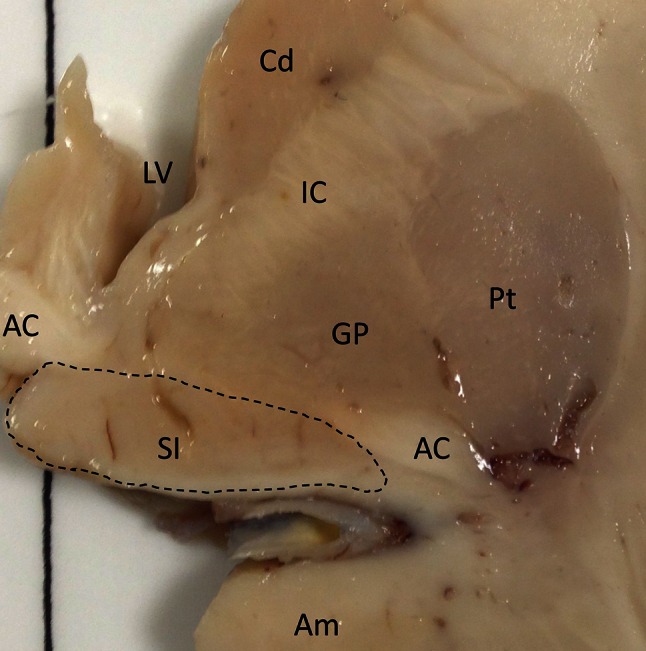
A diagram of the human basal forebrain illustrating the location of the substantia innominata (as outlined). *AC* anterior commissure, *Am* amygdala, *Cd* caudate, *GP* globus pallidus, *IC* internal capsule, *LV* lateral ventricle, *Pt* putamen, *SI* substantia innominata

The basal forebrain region located above and parallel to the optic nerve, with the medial boundary being the wall of the lateral ventricle was first described by Reil in 1809 as the unnamed medullary substance (Die ungenannte Marksubstanz) [84] (Fig. 1). This region was named substantia innominata (SI) of Reil by Theodore Meynert [71]. However, the SI that we commonly refer to is actually more poorly defined anatomically and is known as the SI of Reichert. This is an unlabelled area with no boundaries within Reichert’s human brain atlas from the 1850s [27]. This region has evolved to be known as the anterior perforated substance by Beccari and in a more modern human stereotaxic brain atlas by Schaltenbrand and Bailey simply as basalis to describe the sub-commissural region dorsal to the amygdala [88]. Despite the change in terminology, many current investigators still refer to the region as the SI. However, instead of labelling a region, some investigators describe the SI as discrete group of magnocellular neurons within the basal forebrain synonymous to the nbM we know today [104], reflecting a confusion of terminology in this area.

## Defining a “nucleus”

As mentioned above, Reil was the first to recognise the distinct group of basal forebrain neurons and labelled it as a “medullary substance”. In fact, Meynert described the group of cells as the ganglion of the ansa peduncularis (*ganglion der Hirnschenkelschlinge*), which is found within the SI of Reil bound by the ansa lenticularis dorsally, the optic tract ventrally and the external capsule laterally [71]. Koelliker coined the term ‘basal ganglion of Meynert’ (*Meynert’sches Basalganglion*) and extended Meynert’s finding to describe the ganglion in its rostrocaudal extent [59]. This extends from the mammillary bodies posteriorly to the floor of the inter-hemispheric fissure anteriorly. This ganglion was later called the ganglion of the ansa lenticularis by Edinger (Reviewed in [77]). However, two problems arose from this terminology. First, the term “ganglion” should be used to describe a collection of cell bodies in the peripheral nervous system instead of the central nervous system. Thus, this collection of cells was more closely known as nucleus of the septal plane (*Nucleo del piano settale*) by Beccari [43] and nucleus of the ansa lenticularis by Ayala [8]. Second, the structures ansa peduncularis and ansa lenticularis were difficult to define and the collection of cells was more closely related to the former [77]. Thus, nomenclature associating these neurons to a particular structure was avoided and the term nucleus basalis or basal nucleus (*Der Basalkern* by Brockhaus) was established [15].

## Subdividing the “nucleus”

The nbM is an “open” nucleus with no distinct boundaries and it forms several clusters within the basal forebrain. Attempts have therefore been made to subdivide this ‘nucleus’. Ayala observed two distinct clusters of magnocellular neurons, the first being the previously described nbM and the second located lateral to the anterior commissure and ventral to the putamen for which he coined the term nucleus subputaminalis (NSP) [8]. The NSP is also known as Ayala’s nucleus and it was proposed to be involved in speech function but there is currently no direct evidence to support this hypothesis [92]. Later, Brockhaus also tried to subdivide the nbM and he classified the more anterior part as the pars diffusa and a posterior portion as pars compacta [15].

## nbM: the cholinergic nucleus

In the 1970s, retrograde horseradish peroxidase (HRP) tracer experiments on subhuman primates identified that cortical cholinergic innervation originates from the nbM [68]. Using histochemical and immunohistochemical labelling for acetylcholinesterase (AChE) and choline acetyltransferase (ChAT), Mesulam and colleagues [69, 70] were able to identify the various cholinergic loci in the subhuman primates’ basal forebrain and introduced the nomenclature Ch1–Ch4 to describe four cholinergic cell groups rostrocaudally, with the cholinergic component of the nbM designated as Ch4 (Table 1).

**Table 1:** Basal forebrain cholinergic cell groups and their projections in the brain [69]

Cholinergic group	Region	Projection
Ch1	Medial septal nucleus	Hippocampal complex
Ch2	Vertical limb of the diagonal band nucleus	Hippocampal complex
Ch3	Horizontal limb of the diagonal band nucleus	Olfactory bulb
Ch4	Nucleus basalis of Meynert	Cortex and amygdala

## Cholinergic topographical projection of the nbM

Mesulam and colleagues [69] found that over 90 % of the magnocellular neurons in the nbM are cholinergic and that the Ch4 group is the largest out of the four basal forebrain cholinergic groups. In humans, Ch4 is measured 13–14 mm antero-posteriorly and 16–18 mm medio-laterally within the SI [67]. Furthermore, the Ch4 can be subdivided into five groups in monkeys [69]—the anterior part (Ch4a) into anteromedial (Ch4am) and anterolateral (Ch4al); the intermediate part (Ch4i) into intermediodorsal (Ch4id) and intermedioventral (Ch4iv); and a posterior group (Ch4p). However, there is an additional sixth subsector of the Ch4 in human as the transition between the anterior and intermediate part is elongated, giving rise to the anterointermediate (Ch4ai) region [67]. Prior to this classification, most studies involving the nbM stopped at the level of Ch4i, neglecting the caudal extension. In fact, according to Meynert’s original description, the nbM is located at the plane of the intermediate Ch4 region.

**Fig. 2:**
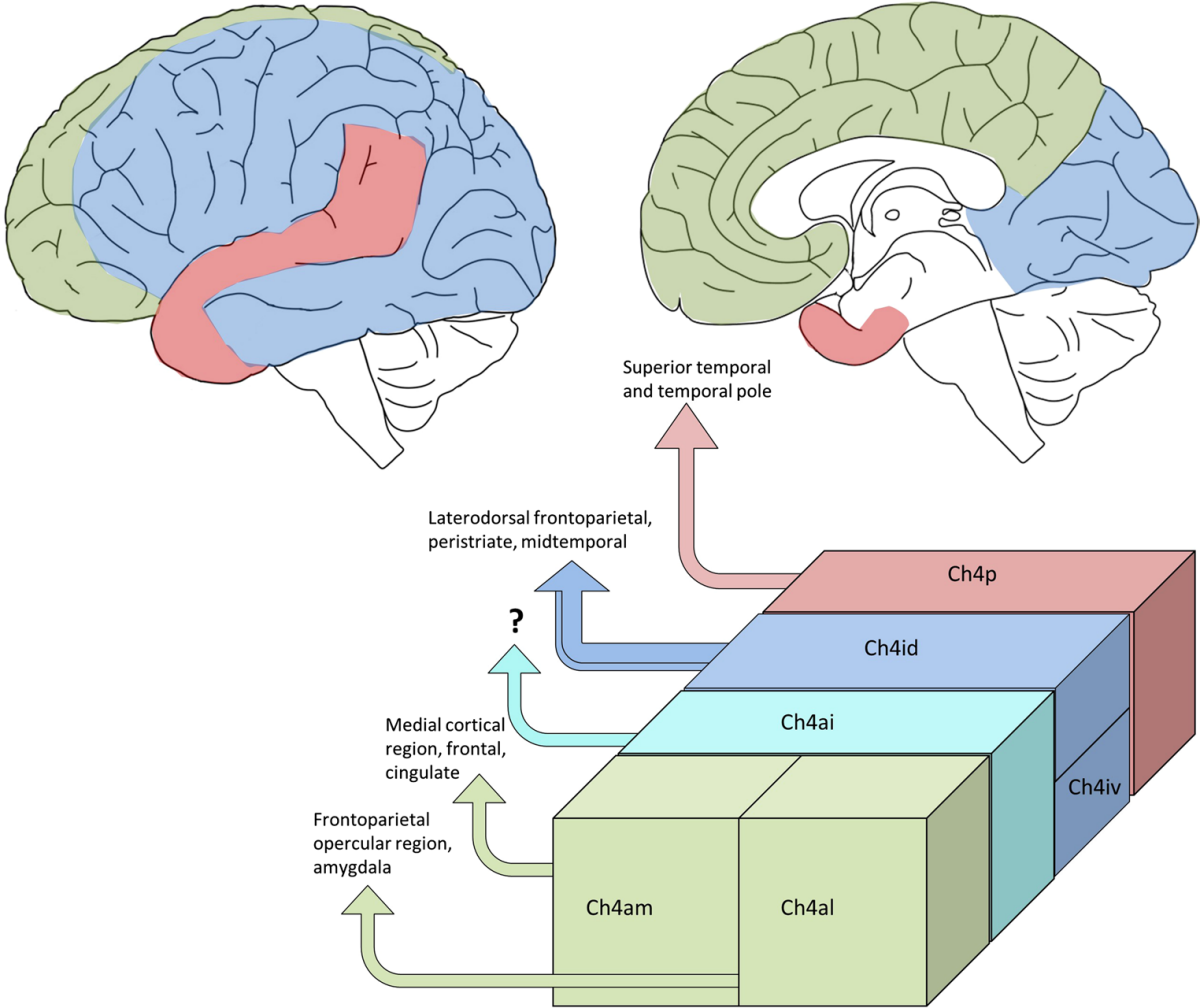
Projected innervation map of the various Ch4 regions (Ch4a, *green*; Ch4i, *blue*; Ch4p, *red*) in the human brain on the lateral surface (*top left*) and at the mid-sagittal plane (*top right*). Cortical projection from the Ch4ai (*turquoise*) is currently unknown in the human brain. Topographical innervation in different subsectors of the nbM according to Mesulam et al. (*bottom*) [67, 69]

Through HRP retrograde tracer and AChE co-localisation studies on macaques, the cortical topographical innervations from the Ch4 subgroups have been mapped out (Fig. 2) [69]. In summary, the anterior Ch4 innervates the limbic regions—Ch4am projects to medial cortical regions including the cingulate cortex and Ch4al projects to fronto-parietal opercular regions and amygdala; Ch4p projects to superior temporal and temporal polar regions; and Ch4i to the remaining cortical regions. It is not known whether these innervation patterns are similar to those in human brain but detailed clinicopathological studies relating to the subdivision of the nbM could provide some clues.

## Problems with the Ch4 subsectors

As pointed out by Mesulam et al. [69], the basal forebrain cholinergic groups do not have strict anatomical boundaries and overlap considerably, in line with the concept that the nbM is an ‘open’ structure rather than a discrete nucleus. Furthermore, within the Ch4 group, some ChAT-immunopositive cells were scattered in different interstitial locations including the anterior commissure, inter-medullary laminae of the globus pallidus, internal capsule, ansa lenticularis and ansa peduncularis. Also, not all magnocellular neurons within the basal forebrain are cholinergic and the terms nbM and Ch4 are therefore not interchangeable.

Ch4ai is a region unique to the human brain, as it is the “gap” between the Ch4a and Ch4i subsectors when results from subhuman primates were translated to humans. It could be speculated that this region is the caudal extension of Ch4a and the rostral extension of Ch4i due to the larger lateral surface area of the neocortex in human as compared with subhuman primates.

Ch4a and Ch4i were further divided into two clusters according to Mesulam. In Ch4a, a vessel or a rarefaction divides it into the medial and lateral sectors. However, with anatomical variation, a vascular structure might not be present in certain planes [47, 87]. Also, the sizes of Ch4am and Ch4al seem not to stay constant throughout [92]. As one progress rostrocaudally, Ch4am appears and overlaps with Ch2 diagonal band nucleus. Then it gradually decreases in size while Ch4al enlarges and merges with Ch4ai. More caudally, the tract of ansa peduncularis is usually an anatomical landmark that divides the Ch4i into dorsal and ventral subsectors, although it can be difficult to identify on thin nbM sections [28, 47]. However, the projection pattern of Ch4id and Ch4iv appears to be similar [69], so the Ch4i can effectively be considered as a single entity.

## Establishment of a simplified nbM subdivisional scheme

**Fig. 3:**
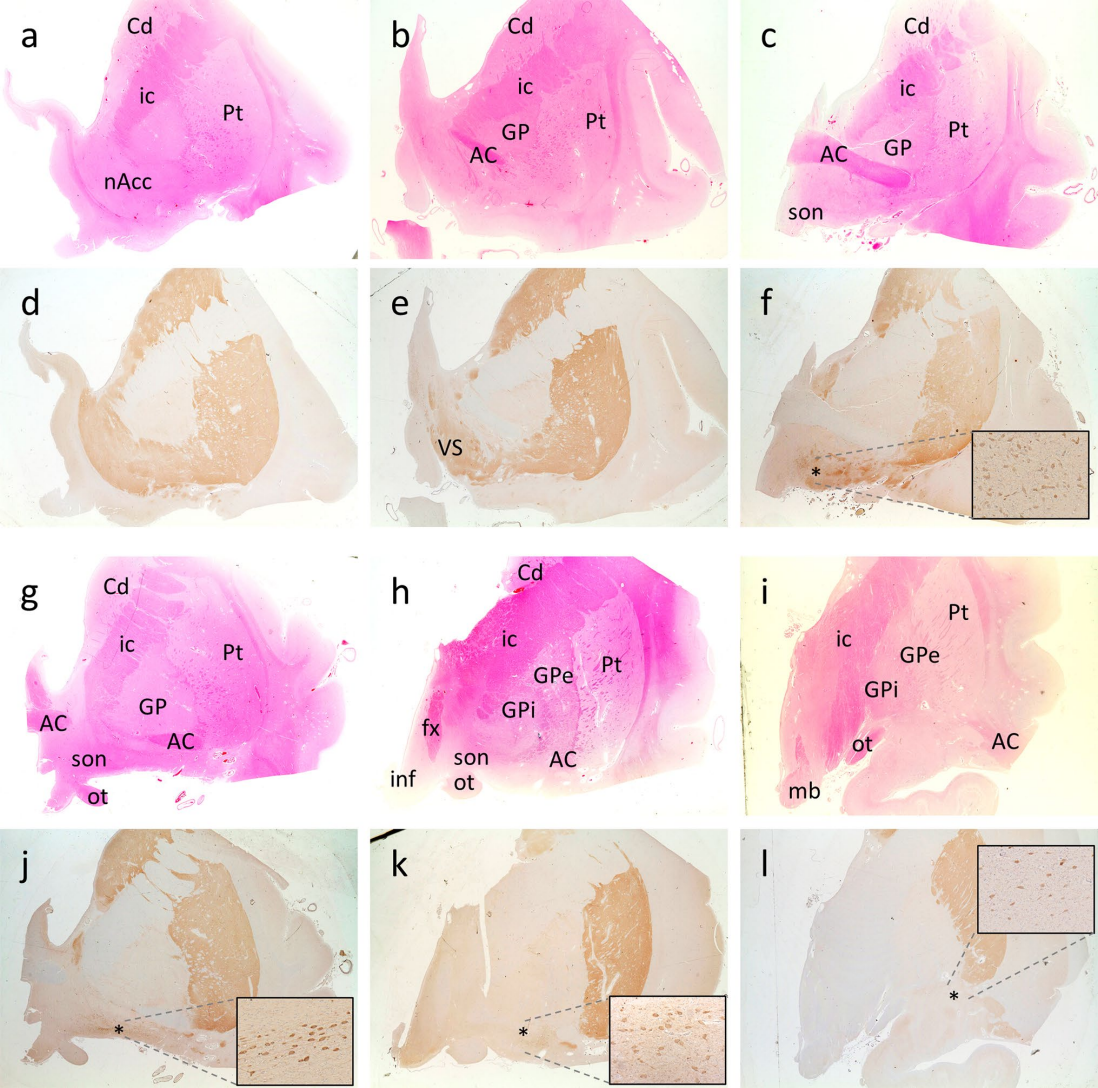
Formalin-fixed, paraffin-embedded basal forebrain sections available from the Parkinson’s UK Tissue Bank at Imperial College, London, stained with H&E (**a**–**c**; **g**–**i**) and serial sections stained with choline acetyltransferase (ChAT) immunohistochemistry (Millipore AB144P, 1:100 with pressure cooker pretreatment in pH 6.0 citrate buffer) (**d**–**f**; **j**–**l**). Six subdivisions of the basal forebrain were defined and arranged rostrally (*top left*) to caudally (*bottom right*). **a**, **d** Level at nucleus accumbens. This level is defined by the absence of anterior commissure and the presence of a large caudate head with nucleus accumbens. **b**, **e** Pre-anterior nbM level. Anterior commissure appears in this section but it is located ventral to the globus pallidus and is rostral to decussation level. A large ventral striatum could be seen clearly with ChAT immunohistochemistry. **c**, **f** Most rostral anterior nbM level. This level is defined by the decussation of the anterior commissure. Ch4 neurons are defined by their location being lateral to the supraoptic nucleus and they are orientated at the medial–lateral axis parallel to the basal border of the section. **g**, **j** Most caudal anterior nbM level. The anterior commissure is split into two parts with medial end still decussating and lateral end located ventral to the globus pallidus. **h**, **k** Intermediate nbM level. At this level, the globus pallidus is split into the external and internal components by an inter-medullary lamina. The anterior commissure is located ventral to the putamen and sometimes the infundibulum could be seen. **i**, **l** Posterior nbM level. This is defined by the presence of mammillary body, small or absence of caudate and internal capsule occupying the medial half of the tissue. *Asterisk* denotes area of maximal density of ChAT-immunopositive cells in the nbM. Zoomed-in figure showing the ChAT-immunopositive neurons in the nbM at ×10 objectives. *AC* anterior commissure, *Cd* caudate, *fx* fornix, *GP* globus pallidus, *GPe* globus pallidus externa, *GPi* globus pallidus interna, *ic* internal capsule, *inf* infundibulum, *mb* mammillary body, *nAcc* nucleus accumbens, *ot* optic tract, *Pt* putamen, *son* supraoptic nucleus, *VS* ventral stratum

**Fig. 4:**
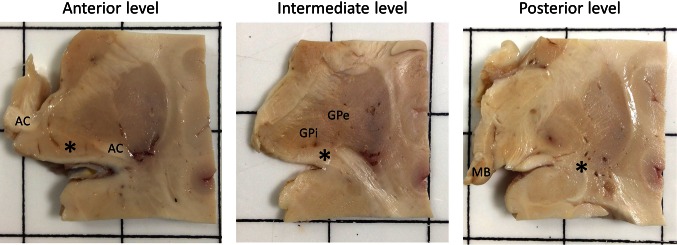
Photographs showing the anatomical landmarks for the anterior, intermediate and posterior levels of the nucleus basalis of Meynert (nbM, as indicated by *asterisk*). At dissection, the first coronal slice is made through the mammillary body (MB), revealing the posterior nbM. Using a 0.5-cm cutting guide two further coronal slices will reveal the intermediate and anterior levels. These are specifically identified by the presence of discernible globus pallidus externa (GPe) and interna (GPi), and midline anterior commissure (AC), respectively. With normal anatomical variation between individuals, this general 0.5 cm interval may need slight modification, depending on brain size

Analysis of the entire nbM will not be possible in many studies, and thus a subdivisional scheme could be useful to simplify and standardise future work on the nbM. We have reviewed several human brain atlases and previous publications [10, 36, 67, 69, 83, 92, 104] and have elaborated a notional definition of anterior, intermediate and posterior subsectors of the nbM (Table 2). The subdivisions we propose approximately correspond to the original Ch4 classification according to Mesulam, discarding the Ch4ai label with its lack of any reliable topographical correlate. This is depicted histologically with H&E and immunohistochemical staining with choline acetyltransferase (ChAT) on the human basal forebrain (Fig. 3). A protocol for recommended sampling at autopsy is given in Fig. 4.

**Table 2:** Proposed macroscopic and microscopic landmark for the definition of anterior, intermediate and posterior subsectors of the nbM

Region	Macroscopic landmark	Microscopic landmark	Corresponding Ch4 regions
Anterior nbM	AC at midline and subpallidal region (continuous or split)	Preoptic or supraoptic nucleus	Ch4am, Ch4al
Intermediate nbM	Globus pallidus divided into GPe and GPi AC ventral to putamen (and GPe)	Supraoptic nucleus, periventricular nucleus, anterior hypothalamic nucleus, (ansa peduncularis)	Ch4al, Ch4i
Posterior nbM	Tail end of AC ventrolateral to putamen Level of mammillary body		Ch4i, Ch4p

## The cholinergic hypothesis and the increased emphasis on nbM research

In the 1970s and 80s, a number of animal and human studies pointed to the importance of acetylcholine in cognition. Subsequently, the “cholinergic hypothesis” was proposed suggesting that a cortical cholinergic deficit leads to cognitive decline in ageing and Alzheimer’s disease (AD) [9]. As the source of cortical cholinergic innervation, the nbM became one of the ‘hot topics’ in dementia research in the last two decades of the 20th century.

## Alzheimer’s disease: a caudorostral pattern of neuronal loss in the nbM

Since Whitehouse and colleagues’ first account of 90 % nbM cell loss in a familial AD case [99], further studies have reported anything from 8 to 87 % cell loss in AD relative to controls [2, 4–6, 17, 19, 25, 29, 46, 47, 64, 65, 73, 74, 78–81, 85, 86, 94, 100–102, 105]. The reason for such a large variation between studies could relate to varying disease severity, but one of the main causes is that neuronal loss is not homogenous throughout the nbM. Therefore, we reviewed the early reports on the neuropathological correlations of nbM in AD with particular emphasis on the region of nbM sampled. Using the aforementioned guideline to divide the nbM into the anterior, intermediate and posterior subdivisions, we identified the regional susceptibility to neuronal loss in AD (Table 3). It appears that in AD, there is a caudorostral gradient of nbM neuronal loss with the posterior sector being the most severely affected. As the posterior nbM contains Ch4p providing cholinergic innervation to the temporal pole and superior temporal cortex [69], this correlates well with memory loss and language impairment in AD.

**Table 3:** Studies of the nbM in AD. Studies where a caudorostral pattern of nbM neuronal loss is found are indicated by [●]

Alzheimer’s disease					
Study	*n*	% Loss relative to control			
		Anterior nbM	Intermediate nbM	Posterior nbM	Not specified/Entire nbM
Whitehouse et al. [99]^a^	1				90 % (Ch4)
Perry et al. [81]	6	33 %			
Whitehouse et al. [100]	5		73 % (Max density) 79 % (Mean density)		
Arendt et al. [4]	14		54 % (Max density) 71.7 % (Mean density)		
Candy et al. [17]	5	35 %			
Nagai et al. [75]	3				66 % (Ch4)
Tagliavini and Pilleri [94]	9		63 %		
Wilcock et al. [102]	6				49 % (Ch4a or Ch4i)
Mann et al. [64]	22		58.9 %		
McGeer et al. [65]	6				33-69.7 % (Ch4)
● Arendt et al. [5]	5	46 % (Ch4am) 51.4 % (Ch4al)	62.6 %	64.2 %	57 % (Ch1–Ch4)
Perry et al. [79]^b^	8				8 % (Moderate AD)
● Rogers et al. [86]	3	64.7 % (Total cell count/section) 44.4 % (Max density)	74.9 % (Total cell count/section) 66.4 % (Max density)		
Doucette et al. [25]	8	50 % (Moderate AD) 70 % (Severe AD)	n.s. (Moderate AD) 65 % (Severe AD)	n.s. (Moderate AD) 80 % (Severe AD)	
● Etienne et al. [29]	10	73 %	87 %		
Rinne et al. [85]	7	22 % 64 %			
Allen et al. [2]	7				61 % (Nissl; Ch4i or Ch4p) 29 % (Neuron specific enolase staining; Ch4i or Ch4p)
Chan-Palay et al. [19]c	12				24 % (Ch4a or Ch4i)
● Wilcock et al. [101]	13	13 % (n.s.) (Ch4am) 52 % in (Ch4al)	41 %	57 %	
● Mufson et al. [73]^d^	7	35.1 % in (Ch4am) 76.4 % in (Ch4al)	62.1 %	76.5 %	
Iraizoz et al. [47]	6	43 %	25 %	30.5 %	31 % (Ch4)
Perry et al. [80]	4				39.3 % (Not specified)
Arendt et al. [6]	64				63.7–86.6 % (Ch4)
Iraizoz et al. [46]	21	48 %	40 %	56 %	40 % (Ch4)
Zarow et al. [105]	86		41.1 %		
	Range	13–76.4 %	25–87 %	30.5–80 %	8–86.6 %

*n.s.* Not significant

^a^Familial AD

^b^Moderate AD

^c^Poor definition of nbM region. According to diagram in the study, area included indicates Ch4a and Ch4i

^d^NGFR immunohistochemistry as marker for cholinergic neurons in the nbM

This pattern of cell loss was supported by some studies where the entire nbM was examined [5, 73, 101]. However, Doucette and colleagues reported that in moderate AD, the anterior nbM had a 50 % neuronal loss while the decrease in cell number was not significant in intermediate and posterior nbM [25]. Similar findings were reported by Iraizoz’s group where the greatest decline was found in anterior followed by posterior nbM [47]. The disagreement concerning the caudorostral pattern of nbM loss in AD could be due to differences in the criteria used to define an nbM neuron for cell counting, as nbM neuronal shrinkage has also been reported in AD [97]. Also, in some of the studies where sections slightly rostral to the anterior commissure decussation were taken, the distinction between Ch4a and Ch2 could be difficult to define. As Ch2 neurons provide innervation to the hippocampus, which is severely affected in AD, the greater loss in the anterior sector of the nbM could be due to the loss of Ch2 rather than Ch4a neurons, although Ch1/2 cell loss in AD has been reported to be minimal [62, 72] or even insignificant [32] compared with age-matched controls.

## Revisiting the nbM in Parkinson’s disease

As mentioned previously, cell loss in the nbM was first identified in PD by Lewy, early in the 20th century. However, quantification of neuronal loss was not attempted until the 1980s where studies reported up to 80 % depletion in the nbM of PD cases (Table 4). When directly comparing PD and AD cases, the loss is comparable [86] or more extensive in PD than in AD [17, 20, 79] and the loss was more apparent among PD with dementia (PDD) cases. Therefore, it is perhaps not be surprising that PDD patients have good neuropsychiatric responses to anticholinesterase medication such as rivastigmine and galantamine (review by [1]). Furthermore, recent imaging studies using cholinergic makers to label acetylcholinesterase have reported significant cortical cholinergic deficits in PD and PDD patients [11–13, 44, 60, 90, 91]. This suggests that apart from the dopaminergic deficit, a decrease in cholinergic tone also contributes to cognitive impairment in PD, as supported by the dual syndrome hypothesis where executive dysfunction and visuospatial impairment in PD correspond to dopaminergic and cholinergic deficits, respectively [53].

**Table 4:** Studies of the nbM in PD

Parkinson’s disease					
Study	*n*	% Loss relative to control			
		Anterior nbM	Intermediate nbM	Posterior nbM	Not specified/entire nbM
Arendt et al. [4]^a^	5		87 % (Total count) 70 % (Max density)		
Candy et al. [17]	3				Greater loss in PD than AD
Whitehouse et al. [98]	4		33.4 % (n.s.)		
Gaspar and Gray [34]	14	32 % (Mean cell count) 34 % (Mean density)			
Nakano and Hirano [76]	11		60.0 % (Mean density) 52.2 % (Max density)		
Tagliavini et al. [95]	3		39.8 %		
Perry et al. [79]	4				17.3 % (Ch4)
Rogers et al. [86]	1	39.8 % (Total cell count/section) 19.0 % (Max density)	39.7 % (Total cell count/section) 32.8 % (Max density)		
Chan-Palay et al. [19]	3				7.7 % (Ch4a or Ch4i)
Perry et al. [80]	7				67.9 % (Not specified)
Zarow et al. [105]^a^	19		37.3 %		
	Range	19.0–39.8 %	32.8–87 %	–	7.7–67.9 %

*n.s.* Not significant

^a^Did not distinguish PDD from PD

The cognitive picture of PDD is commonly considered as a “subcortical” type since patients typically present with dysexecutive signs without significant impairment in storage memory as in “cortical” AD-type dementia [20, 23]. Hence, with varying cortical regions affected in PD and AD the differing clinical profiles may correlate with neuronal loss in particular nbM subsectors. However, only a small number of studies have investigated PDD separately and the different subregions within the nbM have not been compared in PDD cases. We reviewed the literature, estimated the regions sampled in various studies as mentioned before (Table 5) and found a slightly greater deficit in the intermediate nbM region. This supports a recent imaging study [58] which reported a posterior–anterior gradient of cortical cholinergic deficit and this could be due to the extensive cell loss in the Ch4i affecting the cholinergic innervation to occipital–parietal cortical regions.

**Table 5:** Studies of the nbM in PDD

Parkinson’s disease dementia					
Study	*n*	% Loss relative to control			
		Anterior nbM	Intermediate nbM	Posterior nbM	Not specified/entire nbM
Whitehouse et al. [98]	2		77.1 % (Total cell count) 53.1 % (Max density)		
Gaspar and Gray [34]	18	60 % (Mean cell count) 58 % (Mean density)			
Perry et al. [79]	10				72.2 % (Ch4; PDD and DLB not distinguished) 74.7 % (Ch4; PD with AD)
Rogers et al. [86]	3	44.3 % (Total cell count/section) 36.5 % (Max density)	72.3 % (Total cell count/section) 62.2 % (Max density)		
Chui et al. [20]	3	66.1 %			
Chan-Palay et al. [19]	6				47.4 % (Ch4a or Ch4i)
Perry et al. [80]	14				41.7 % (Ch4; PDD and DLB not distinguished)
	Range	36.5–66.1 %	53.1–77.1 %	–	41.7–74.7 %

## A dichotomous pattern of nbM cell loss in PD and AD

Amyloid-beta (Aβ) plaques and tau neurofibrillary tangles (NFT) are hallmark neuropathological features of AD, the latter being more closely associated with cognitive decline [7]. It has been well recognised that LB- and AD-type pathologies frequently co-exist in the brains of PDD and dementia with Lewy bodies (DLB) [49] and that there may be synergistic relationships between the two types of pathologies in the development of dementia [21, 48].

Cullen and Halliday [22] proposed that the cause of neuronal loss in the nbM could differ between PD and AD. They studied cell loss and NFT pathology within the nbM of AD and LB disease with concomitant AD. Severe cell loss in the nbM of AD cases was accompanied by abundant extraneuronal NFT pathology. However, cases of LB disease with AD have equally severe nbM depletion, despite the relatively milder NFT pathology. This suggests that the alpha-synuclein pathology could also be involved in nbM neuronal death in cases with LB pathology. This dichotomous disease process affecting the nbM in PD and AD was also described by Candy and colleagues. They reported that nbM neuronal loss in PD was more extensive than in AD in the absence of co-existing cortical NFT pathology [17]. Similarly, Gaspar and Gray noted that in 5 of 6 PDD cases, there was severe nbM neuronal depletion despite relatively little or no cortical AD-type pathology [34]. They concluded that cortical AD pathologies did not seem to affect the reduction of cholinergic cortical afferents in PD. In addition, Nakano and Hirano reported that neuronal loss in the nbM of PD is not associated with NFT in the cortex, hippocampus or in the nbM [76]. Therefore, in order to study nbM loss in ‘pure’ PD, cases with severe AD pathologies should be excluded. In the studies we reviewed (Tables 4, 5), most [17, 19, 20, 34, 79, 80, 95] but not all [4, 86, 98, 105] have excluded cases with severe co-existing AD pathologies.

Striatal Aβ has been suggested to contribute to the development of dementia in PD and DLB, independently of comorbid AD pathologies [51, 52]. Although a couple of studies reported striatal Aβ as specific to DLB not PDD [39, 50], and subsequently concluded Aβ load in the striatum affects the temporal relationship between dementia and PD motor symptoms rather than presence of dementia, controversies remain as to whether the severity of striatal Aβ could differentiate PDD from DLB.

Other basal forebrain cholinergic nuclei such as the medial septal nucleus (Ch1) and the vertical limb of the diagonal band nucleus (Ch2) also show differential susceptibility in AD and LB disorders. Fujishiro and colleagues reported the loss of Ch1 and Ch2 ChAT-positive neurons in DLB but not AD cases compared with controls [32]. However, a recent study reported no significant change in Ch1 and Ch2 ChAT-positive neurons in PD and PDD [38]. So further work is needed to identify the potentially subtle pathological differences between PDD and DLB. One further consideration, as mentioned previously, is that other neurotransmitter deficits could contribute to cognitive decline in PD. Cell loss in the nbM happens in parallel with dopaminergic neuronal loss in the substantia nigra and ventral tegmental area; and noradrenergic neuronal loss in the locus coeruleus [54]. Hence, the basal forebrain depletion could also be associated with the decrease in dopaminergic and noradrenergic innervation in PD. Finally, serotoninergic dysfunction, dysregulation of excitatory amino acid and purinergic interactions in PD [93] should not be neglected as they might also contribute to non-motor symptoms including cognitive impairment in PD.

## Possible functional correlate to subdivisional neuronal loss in the nbM in PD

Correlation relating specific cognitive deficits in PD with subsector pathology in the nbM has not been achieved, in part because of a lack of detailed neuropsychological testing in extant postmortem brain studies. An exception is from one study by Chui and co-workers in which three neuropathologically confirmed PD cases with dementia had undergone extensive neuropsychiatric tests before death and detailed cognitive profiles for the patients were available [20]. Their cognitive impairment included the presence of hallucination, visuospatial impairment and attentional deficits typical in PD cases. When the cell count of nbM (apparently Ch4a) was compared against AD cases, a greater decrease in mean density was observed in PDD (66.1 % loss) than in AD (61.9 % loss). On the other hand, the correlation between cognitive impairment in LB disorders and regional cortical involvement has been well supported by various functional imaging studies. In particular, atrophy and hypometabolism in the occipito-parietal region and, to a lesser extent, the frontal cortex in PDD patients relative to controls have been reported [35]. One study has compared the specific cognitive impairment in PD with regional fluorodeoxyglucose (FDG) uptake [33]. The degree of executive dysfunction in PD patients correlated positively to hypometabolism score in the frontal lobe, whereas visuospatial function impairment correlated to occipito-parietal reduction in FDG uptake.

Hence, one could speculate that pathology in Ch4a correlates with executive dysfunction in PDD due to frontal and limbic cortical innervation from the anterior Ch4 area. Moreover, anosmia in PD and PDD has been shown to be associated with limbic cortical cholinergic denervation, which could again correlate with Ch4a pathology [14]. Similarly, visuospatial impairment in PDD or even in early PD would possibly be due to neuronal loss in the Ch4i subsector. As PD/PDD patients typically have a less amnestic profile than AD, we would expect Ch4p to be relatively spared. However, visual hallucination in PD is a more complex phenomenon which might be due to a combination of occipito-parietal hypometabolism [45] and the presence of LB in the temporal lobe [40]. Therefore, pathology in the Ch4i and Ch4p regions may play a role in this characteristic element of cognitive dysfunction in PDD/DLB, along with other brain centres.

In addition, in earlier studies, no distinction was made between PDD and DLB [79, 80], as DLB is a more recently established clinical entity [66], and it would be of interest to investigate whether the pattern of cell loss within the nbM subregions differs in PDD and DLB.

## Conclusion and future work

**Fig. 5:**
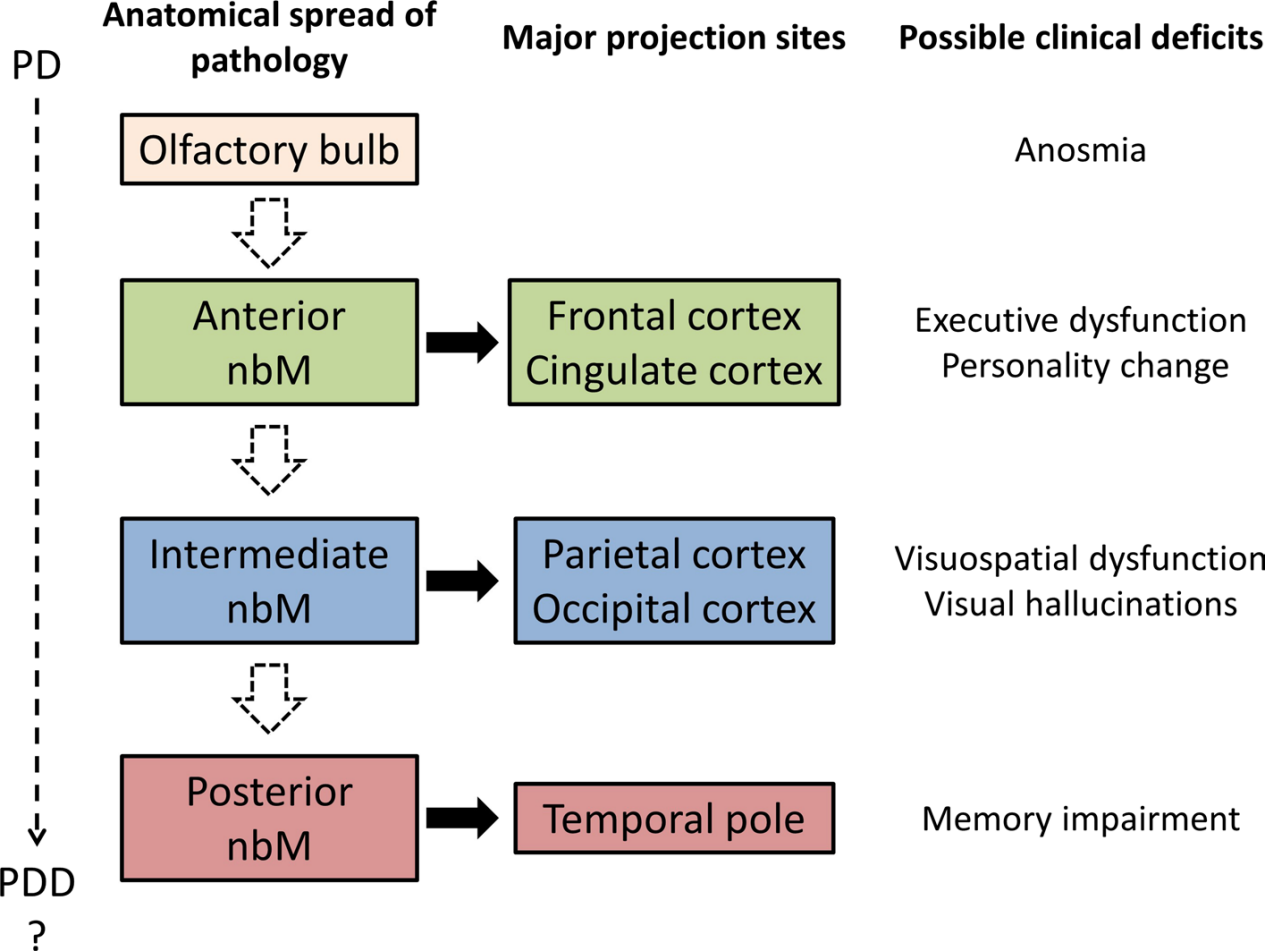
Projected schema of anatomical progression of pathology within the nbM with possible clinicopathological correlations. Hypothesised progression is indicated by *dashed arrows*

Following our literature review on the studies of the nbM above, we also revisited the original work by Friedrich Lewy [30] and found that the area he defined as the nbM was from the optic tract to septum pellucidum. This would equate to the anterior/intermediate Ch4 region according to modern classification and thus historical evidence illustrates that LB and severe neuronal depletion were first described in the anterior portion of nbM. Along with the collective evidence and our speculation that there is a relative sparing of posterior nbM involvement in PD patients, it could be hypothesised that pathology in the nbM begins in the anterior portion and progresses caudally in PD. This anatomical progression supports the prion-like propagation hypothesis [3, 16, 26, 31] and the dual-hit hypothesis of alpha-synuclein [41, 42] where pathology starts in the olfactory bulb and spreads towards the basal forebrain region (Fig. 5). However, further studies investigating the topographical innervation pattern from different subsectors of the human nbM to target regions and reciprocal connectivity are needed, particularly with the advancement in tractography and other high-resolution imaging techniques.

With the increasing number of imaging studies focusing on basal forebrain changes [56, 57] there is a need for this nucleus to be revisited in pathological studies. The potential for neuromodulatory treatment targeting the nbM is now being realised, in particular deep brain stimulation in dementia [37, 61] and stereotactic gene delivery of trophic factors [82]. However, it is important to note that there are many caveats to consider, including distinctly varying pathogenesis of dementia in PD and AD. Better clinicopathological correlations have to be established, especially in relation to the different subregions of the nbM. This will improve our understanding of the pathological basis for different forms of dementing disorders and the role of forebrain cholinergic mechanisms in normal cognition as well as in the setting of cognitive decline.

## References

[1] Aarsland D, Mosimann UP, McKeith IG (2004). Role of cholinesterase inhibitors in Parkinson’s disease and dementia with Lewy bodies. J Geriatr Psychiatry Neurol.

[2] Allen SJ, Dawbarn D, Wilcock GK (1988). Morphometric immunochemical analysis of neurons in the nucleus basalis of Meynert in Alzheimer’s disease. Brain Res.

[3] Angot E, Steiner JA, Hansen C (2010). Are synucleinopathies prion-like disorders?. Lancet Neurol.

[4] Arendt T, Bigl V, Arendt A, Tennstedt A (1983). Loss of neurons in the nucleus basalis of Meynert in Alzheimer’s disease, paralysis agitans and Korsakoff’s Disease. Acta Neuropathol.

[5] Arendt T, Bigl V, Tennstedt A, Arendt A (1985). Neuronal loss in different parts of the nucleus basalis is related to neuritic plaque formation in cortical target areas in Alzheimer’s disease. Neuroscience.

[6] Arendt T, Schindler C, Brückner MK (1997). Plastic neuronal remodeling is impaired in patients with Alzheimer’s disease carrying apolipoprotein epsilon 4 allele. J Neurosci.

[7] Arriagada PV, Marzloff K, Hyman BT (1992). Distribution of Alzheimer-type pathologic changes in nondemented elderly individuals matches the pattern in Alzheimer’s disease. Neurology.

[8] Ayala G (1915). A hitherto undifferentiated nucleus in the forebrain (nucleus subputaminalis). Brain.

[9] Bartus RT, Dean RL, Beer B, Lippa A (1982). The cholinergic hypothesis of geriatric memory dysfunction. Science.

[10] Boban M, Kostovic I, Simic G (2006) Nucleus subputaminalis: neglected part of the basal nucleus of Meynert. Brain 129:E42; author reply E43. doi:10.1093/brain/awl025 10.1093/brain/awl025 16543395

[11] Bohnen NI, Kaufer DI, Hendrickson R (2006). Cognitive correlates of cortical cholinergic denervation in Parkinson’s disease and parkinsonian dementia. J Neurol.

[12] Bohnen NI, Müller MLTM, Koeppe RA (2009). History of falls in Parkinson disease is associated with reduced cholinergic activity. Neurology.

[13] Bohnen NI, Kaufer DI, Ivanco LS (2003). Cortical cholinergic function is more severely affected in parkinsonian dementia than in Alzheimer disease: an in vivo positron emission tomographic study. Arch Neurol.

[14] Bohnen NI, Müller MLTM, Kotagal V (2010). Olfactory dysfunction, central cholinergic integrity and cognitive impairment in Parkinson’s disease. Brain.

[15] Brockhaus H (1942). Vergleichend-anatomische Untersuchungen über den Basalkernkomplex. J Psychol Neurol.

[16] Brundin P, Melki R, Kopito R (2010). Prion-like transmission of protein aggregates in neurodegenerative diseases. Nat Rev Mol Cell Biol.

[17] Candy JM, Perry RH, Perry EK (1983). Pathological changes in the nucleus of Meynert in Alzheimer’s and Parkinson’s diseases. J Neurol Sci.

[18] Casanova MF, Walker LC, Whitehouse PJ, Price DL (1985). Abnormalities of the nucleus basalis in Down’s syndrome. Ann Neurol.

[19] Chan-Palay V (1988). Galanin hyperinnervates surviving neurons of the human basal nucleus of Meynert in dementias of Alzheimer’s and Parkinson’s disease: a hypothesis for the role of galanin in accentuating cholinergic dysfunction in dementia. J Comp Neurol.

[20] Chui HC, Mortimer JA, Slager U (1986). Pathologic correlates of dementia in Parkinson’s disease. Arch Neurol.

[21] Compta Y, Parkkinen L, O’Sullivan SS (2011). Lewy- and Alzheimer-type pathologies in Parkinson’s disease dementia: which is more important?. Brain.

[22] Cullen KM, Halliday GM (1998). Neurofibrillary degeneration and cell loss in the nucleus basalis in comparison to cortical Alzheimer pathology. Neurobiol Aging.

[23] Darvesh S, Freedman M (1996). Subcortical dementia: a neurobehavioral approach. Brain Cogn.

[24] Divac I (1975). Magnocellular nuclei of the basal forebrain project to neocortex, brain stem, and olfactory bulb. Review of some functional correlates. Brain Res.

[25] Doucette R, Fisman M, Hachinski VC, Mersky H (1986). Cell loss from the nucleus basalis of Meynert in Alzheimer’s disease. Can J Neurol Sci.

[26] Dunning CJR, Reyes JF, Steiner JA, Brundin P (2012). Can Parkinson’s disease pathology be propagated from one neuron to another?. Prog Neurobiol.

[27] Engelhardt E (2013). Meynert and the basal nucleus. Dement Neuropsychol.

[28] Etienne P, Robitaille Y, Gauthier S, Nair NP (1986). Nucleus basalis neuronal loss and neuritic plaques in advanced Alzheimer’s disease. Can J Physiol Pharmacol.

[29] Etienne P, Robitaille Y, Wood P (1986). Nucleus basalis neuronal loss, neuritic plaques and choline acetyltransferase activity in advanced Alzheimer’s disease. Neuroscience.

[30] Föorstl H, Levy R (1991). F. H. Lewy on Lewy bodies, parkinsonism and dementia. Int J Geriatr Psychiatry.

[31] Frost B, Diamond MI (2010). Prion-like mechanisms in neurodegenerative diseases. Nat Rev Neurosci.

[32] Fujishiro H, Umegaki H, Isojima D (2006). Depletion of cholinergic neurons in the nucleus of the medial septum and the vertical limb of the diagonal band in dementia with Lewy bodies. Acta Neuropathol.

[33] Garcia-Garcia D, Clavero P, Gasca Salas C (2012). Posterior parietooccipital hypometabolism may differentiate mild cognitive impairment from dementia in Parkinson’s disease. Eur J Nucl Med Mol Imaging.

[34] Gaspar P, Gray F (1984). Dementia in idiopathic Parkinson’s disease. A neuropathological study of 32 cases. Acta Neuropathol.

[35] González-Redondo R, García-García D, Clavero P (2014). Grey matter hypometabolism and atrophy in Parkinson’s disease with cognitive impairment: a two-step process. Brain.

[36] Gorry JD (1963). Studies on the comparative anatomy of the Ganglion Basale of Meynert. Acta Anat (Basel).

[37] Gratwicke J, Kahan J, Zrinzo L (2013). The nucleus basalis of Meynert: a new target for deep brain stimulation in dementia?. Neurosci Biobehav Rev.

[38] Hall H, Reyes S, Landeck N (2014). Hippocampal Lewy pathology and cholinergic dysfunction are associated with dementia in Parkinson’s disease. Brain.

[39] Halliday GM, Song YJC, Harding AJ (2011). Striatal β-amyloid in dementia with Lewy bodies but not Parkinson’s disease. J Neural Transm.

[40] Harding AJ, Broe GA, Halliday GM (2002). Visual hallucinations in Lewy body disease relate to Lewy bodies in the temporal lobe. Brain.

[41] Hawkes CH, Del Tredici K, Braak H (2007). Parkinson’s disease: a dual-hit hypothesis. Neuropathol Appl Neurobiol.

[42] Hawkes CH, Del Tredici K, Braak H (2009). Parkinson’s disease: the dual hit theory revisited. Ann NY Acad Sci.

[43] Heimer L, De Olmos JS, Alheid GF, Floyd E, Bloom A, Bjorklund T (1999). The human basal forebrain. Part II. The primate nervous system, part 3.

[44] Hilker R, Thomas AV, Klein JC (2005). Dementia in Parkinson disease: functional imaging of cholinergic and dopaminergic pathways. Neurology.

[45] Imamura T, Ishii K, Hirono N (1999). Visual hallucinations and regional cerebral metabolism in dementia with Lewy bodies (DLB). Neuroreport.

[46] Iraizoz I, Guijarro JL, Gonzalo LM, de Lacalle S (1999). Neuropathological changes in the nucleus basalis correlate with clinical measures of dementia. Acta Neuropathol.

[47] Iraizoz I, de Lacalle S, Gonzalo LM (1991). Cell loss and nuclear hypertrophy in topographical subdivisions of the nucleus basalis of Meynert in Alzheimer’s disease. Neuroscience.

[48] Irwin DJ, Lee VM-Y, Trojanowski JQ (2013). Parkinson’s disease dementia: convergence of α-synuclein, tau and amyloid-β pathologies. Nat Rev Neurosci.

[49] Jellinger KA, Attems J (2014). Challenges of multimorbidity of the aging brain: a critical update. J Neural Transm.

[50] Jellinger KA, Attems J (2006). Does striatal pathology distinguish Parkinson disease with dementia and dementia with Lewy bodies?. Acta Neuropathol.

[51] Kalaitzakis ME, Walls AJ, Pearce RKB, Gentleman SM (2011). Striatal Aβ peptide deposition mirrors dementia and differentiates DLB and PDD from other parkinsonian syndromes. Neurobiol Dis.

[52] Kalaitzakis ME, Graeber MB, Gentleman SM, Pearce RKB (2008). Striatal beta-amyloid deposition in Parkinson disease with dementia. J Neuropathol Exp Neurol.

[53] Kehagia AA, Barker RA, Robbins TW (2013). Cognitive impairment in Parkinson’s disease: the dual syndrome hypothesis. Neurodegener Dis.

[54] Kehagia AA, Barker RA, Robbins TW (2010). Neuropsychological and clinical heterogeneity of cognitive impairment and dementia in patients with Parkinson’s disease. Lancet Neurol.

[55] Kievit J, Kuypers HG (1975). Basal forebrain and hypothalamic connection to frontal and parietal cortex in the Rhesus monkey. Science.

[56] Kilimann I, Grothe M, Heinsen H (2014). Subregional basal forebrain atrophy in Alzheimer’s disease: a multicenter study. J Alzheimers Dis.

[57] Kim HJ, Lee JE, Shin SJ (2011). Analysis of the substantia innominata volume in patients with Parkinson’s disease with dementia, dementia with Lewy bodies, and Alzheimer’s disease. J Mov Disord.

[58] Klein JC, Eggers C, Kalbe E (2010). Neurotransmitter changes in dementia with Lewy bodies and Parkinson disease dementia in vivo. Neurology.

[59] Koelliker A (1896) Handbuch der Gewebelehre des Menschen. In: Nervensystem des Menschen und der Thiere. vol 2, 6th edn. W. Engelmann, Leipzig

[60] Kuhl DE, Minoshima S, Fessler JA (1996). In vivo mapping of cholinergic terminals in normal aging, Alzheimer’s disease, and Parkinson’s disease. Ann Neurol.

[61] Kuhn J, Hardenacke K, Lenartz D (2014). Deep brain stimulation of the nucleus basalis of Meynert in Alzheimer’s dementia. Mol Psychiatry.

[62] Lehéricy S, Hirsch EC, Cervera-Piérot P (1993). Heterogeneity and selectivity of the degeneration of cholinergic neurons in the basal forebrain of patients with Alzheimer’s disease. J Comp Neurol.

[63] Lewy F (1913). Zur pathologischen Anatomie der Paralysis agitans. Dtsch Z Nervenheilk.

[64] Mann DM, Yates PO, Marcyniuk B (1984). Alzheimer’s presenile dementia, senile dementia of Alzheimer type and Down’s syndrome in middle age form an age related continuum of pathological changes. Neuropathol Appl Neurobiol.

[65] McGeer PL, McGeer EG, Suzuki J (1984). Aging, Alzheimer’s disease, and the cholinergic system of the basal forebrain. Neurology.

[66] McKeith IG, Dickson DW, Lowe J (2005). Diagnosis and management of dementia with Lewy bodies: third report of the DLB Consortium. Neurology.

[67] Mesulam MM, Geula C (1988). Nucleus basalis (Ch4) and cortical cholinergic innervation in the human brain: observations based on the distribution of acetylcholinesterase and choline acetyltransferase. J Comp Neurol.

[68] Mesulam MM, Van Hoesen GW (1976). Acetylcholinesterase-rich projections from the basal forebrain of the rhesus monkey to neocortex. Brain Res.

[69] Mesulam MM, Mufson EJ, Levey AI, Wainer BH (1983). Cholinergic innervation of cortex by the basal forebrain: cytochemistry and cortical coa, diagonal band nuclei, connections of the septal areleus basalis (substantia innominata), and hypothalamus in the rhesus monkey. J Comp Neurol.

[70] Mesulam MM, Mufson EJ, Levey AI, Wainer BH (1984). Atlas of cholinergic neurons in the forebrain and upper brainstem of the macaque based on monoclonal choline acetyltransferase immunohistochemistry and acetylcholinesterase histochemistry. Neuroscience.

[71] Meynert T, Putnam J (translated) (1872) The brain of mammals. In: Stricker S (ed) A Man. Histol. W. Wood & company, New York, pp 650–766

[72] Mufson EJ, Cochran E, Benzing W, Kordower JH (1993). Galaninergic innervation of the cholinergic vertical limb of the diagonal band (Ch2) and bed nucleus of the stria terminalis in aging, Alzheimer’s disease and Down’s syndrome. Dementia.

[73] Mufson EJ, Bothwell M, Kordower JH (1989). Loss of nerve growth factor receptor-containing neurons in Alzheimer’s disease: a quantitative analysis across subregions of the basal forebrain. Exp Neurol.

[74] Nagai T, McGeer PL, Peng JH (1983). Choline acetyltransferase immunohistochemistry in brains of Alzheimer’s disease patients and controls. Neurosci Lett.

[75] Nagai T, Pearson T, Peng F (1983). Immunohistochemical staining of the human forebrain with monoclonal antibody to human choline acetyltransferase. Brain Res.

[76] Nakano I, Hirano A (1984). Parkinson’s disease: neuron loss in the nucleus basalis without concomitant Alzheimer’s disease. Ann Neurol.

[77] Papez JW, Aronson LR (1934). Thalamic nuclei of Pithecus (Macacus) Rhesus. Arch Neurol Psychiatry.

[78] Pearson RC, Sofroniew MV, Cuello AC (1983). Persistence of cholinergic neurons in the basal nucleus in a brain with senile dementia of the Alzheimer’s type demonstrated by immunohistochemical staining for choline acetyltransferase. Brain Res.

[79] Perry EK, Curtis M, Dick DJ (1985). Cholinergic correlates of cognitive impairment in Parkinson’s disease: comparisons with Alzheimer’s disease. J Neurol Neurosurg Psychiatry.

[80] Perry EK, Irving D, Kerwin JM (1993). Cholinergic transmitter and neurotrophic activities in Lewy body dementia: similarity to Parkinson’s and distinction from Alzheimer disease. Alzheimer Dis Assoc Disord.

[81] Perry RH, Candy JM, Perry EK (1982). Extensive loss of choline acetyltransferase activity is not reflected by neuronal loss in the nucleus of Meynert in Alzheimer’s disease. Neurosci Lett.

[82] Rafii MS, Baumann TL, Bakay RA (2014). A phase 1 study of stereotactic gene delivery of AAV2-NGF for Alzheimer’s disease. Alzheimers Dement.

[83] Raghanti MA, Simic G, Watson S (2011). Comparative analysis of the nucleus basalis of Meynert among primates. Neuroscience.

[84] Reil JC (1809). Archiv für die Physiologie.

[85] Rinne JO, Paljärvi L, Rinne UK (1987). Neuronal size and density in the nucleus basalis of Meynert in Alzheimer’s disease. J Neurol Sci.

[86] Rogers JD, Brogan D, Mirra SS (1985). The nucleus basalis of Meynert in neurological disease: a quantitative morphological study. Ann Neurol.

[87] Saper CB, Chelimsky TC (1984). A cytoarchitectonic and histochemical study of nucleus basalis and associated cell groups in the normal human brain. Neuroscience.

[88] Schaltenbrand G, Bailey P (1959) Einführung in die Stereotakischen Operationen Mit Einem Atlas des Menschlichen Gehirns (Introduction to stereotaxis with an atlas of the human brain). vol. 1–3. Georg Thieme, Stuttgart

[89] Schiller F (2000). Fritz Lewy and his bodies. J Hist Neurosci.

[90] Shimada H, Hirano S, Shinotoh H (2009). Mapping of brain acetylcholinesterase alterations in Lewy body disease by PET. Neurology.

[91] Shinotoh H, Namba H, Yamaguchi M (1999). Positron emission tomographic measurement of acetylcholinesterase activity reveals differential loss of ascending cholinergic systems in Parkinson’s disease and progressive supranuclear palsy. Ann Neurol.

[92] Simić G, Mrzljak L, Fucić A (1999). Nucleus subputaminalis (Ayala): the still disregarded magnocellular component of the basal forebrain may be human specific and connected with the cortical speech area. Neuroscience.

[93] Stayte S, Vissel B (2014). Advances in non-dopaminergic treatments for Parkinson’s disease. Front Neurosci.

[94] Tagliavini F, Pilleri G (1983). Basal nucleus of Meynert. A neuropathological study in Alzheimer’s disease, simple senile dementia, Pick’s disease and Huntington’s chorea. J Neurol Sci.

[95] Tagliavini F, Pilleri G, Bouras C, Constantinidis J (1984). The basal nucleus of Meynert in idiopathic Parkinson’s disease. Acta Neurol Scand.

[96] Uhl GR, McKinney M, Hedreen JC (1982). Dementia pugilistica: loss of basal forebrain cholinergic neurons and cholinergic cortical markers. Ann Neurol.

[97] Vogels OJM, Broere CA, Ter Laak HJ (1990). Cell loss and shrinkage in the nucleus basalis Meynert complex in Alzheimer’s disease. Neurobiol Aging.

[98] Whitehouse PJ, Hedreen JC, White CL, Price DL (1983). Basal forebrain neurons in the dementia of Parkinson disease. Ann Neurol.

[99] Whitehouse PJ, Price DL, Clark AW (1981). Alzheimer disease: evidence for selective loss of cholinergic neurons in the nucleus basalis. Ann Neurol.

[100] Whitehouse PJ, Price DL, Struble RG (1982). Alzheimer’s disease and senile dementia: loss of neurons in the basal forebrain. Science.

[101] Wilcock GK, Esiri MM, Bowen DM, Hughes AO (1988). The differential involvement of subcortical nuclei in senile dementia of Alzheimer’s type. J Neurol Neurosurg Psychiatry.

[102] Wilcock GK, Esiri MM, Bowen DM, Smith CC (1983). The nucleus basalis in Alzheimer’s disease: cell counts and cortical biochemistry. Neuropathol Appl Neurobiol.

[103] Williams MR, Marsh R, Macdonald CD (2013). Neuropathological changes in the nucleus basalis in schizophrenia. Eur Arch Psychiatry Clin Neurosci.

[104] Zaborszky L, Hoemke L, Mohlberg H (2008). Stereotaxic probabilistic maps of the magnocellular cell groups in human basal forebrain. Neuroimage.

[105] Zarow C, Lyness SA, Mortimer JA, Chui HC (2003). Neuronal loss is greater in the locus coeruleus than nucleus basalis and substantia nigra in Alzheimer and Parkinson diseases. Arch Neurol.

